# Sexually-dimorphic offspring outcomes following antenatal corticosteroids: a review of the evidence for congenital adrenal hyperplasia and preterm birth risk

**DOI:** 10.1186/s13293-026-00938-1

**Published:** 2026-07-03

**Authors:** Lulu Y. L. Birch, Amanda N. Sferruzzi-Perri

**Affiliations:** Department of Physiology, Loke Centre for Trophoblast Research, Development and Neuroscience, University of Cambridge, Cambridge, CB2 3EG UK

**Keywords:** Congenital adrenal hyperplasia, Preterm birth, Sex differences, Antenatal corticosteroids

## Abstract

**Background:**

Pregnant individuals may receive antenatal corticosteroids for various conditions, including congenital adrenal hyperplasia (to prevent virilisation in female fetuses) and preterm birth risk (to hasten fetal maturation and improve perinatal survival). This narrative review aimed to evaluate the evidence of sexually-dimorphic offspring outcomes following antenatal corticosteroids for such conditions and explore the potential underlying mechanisms.

**Methods:**

A comprehensive MEDLINE search identified twenty-eight studies for inclusion: seven focused on congenital adrenal hyperplasia and twenty-one focused on preterm birth risk.

**Results:**

For congenital adrenal hyperplasia, evidence suggests that antenatal corticosteroids are associated with sexually-dimorphic outcomes across cognition, behaviour and brain structure. Females demonstrated more consistently negative outcomes, particularly within the congenital adrenal hyperplasia-affected population, where females were exposed to longer treatment durations than males. The outcomes for males were more heterogenous. For preterm birth, some studies suggest preterm males remain at increased risk for mortality and morbidity, while in term-born infants, females may have worse outcomes. The nature of these effects, however, differed with antenatal corticosteroid types, and conflicting findings also existed. Potential mechanisms for sexually-dimorphic outcomes include differences in placental enzyme and receptor expression and activity, fetal hypothalamic-pituitary-adrenal (HPA) axis function, and susceptibility to developing respiratory conditions.

**Conclusions:**

The current literature suggests potential sex differences following antenatal corticosteroids, which differ between congenital adrenal hyperplasia and preterm birth risk. Owing to surmounting evidence of negative outcomes and the retracting use of antenatal corticosteroids for congenital adrenal hyperplasia, further research will be challenging. Whereas studies concerning sexually-dimorphic responses to antenatal corticosteroids for preterm birth risk are essential, given their widespread use. Further research in this area may help to understand whether the clinical management of preterm birth should be adjusted according to sex.

## Highlights

Pregnant individuals may be administered antenatal corticosteroids for various conditions, yet whether they may yield sexually-dimorphic outcomes requires study.

This comprehensive literature review revealed that sexually-dimorphic outcomes may indeed follow antenatal corticosteroid administration for both congenital adrenal hyperplasia and preterm birth health risks.

However, the nature of these outcomes depended on the reason for treatment, the corticosteroid type, and was at times conflicting.

Putative mechanisms for sexually-dimorphic outcomes include differences in placental enzyme activity, fetal HPA axis function, and respiratory complications.

## Background

Antenatal corticosteroids are synthetic corticosteroids that bypass the 11beta-hydroxysteroid dehydrogenase type 2 enzyme, allowing passage across the placenta to the developing fetus. Their use was due to seminal findings from Liggins and Howie [3] who demonstrated that administration to pregnant individuals at risk of preterm birth significantly reduced neonatal mortality and morbidity, notably respiratory distress syndrome. Presently, individuals between 24 + 0 weeks and 36 + 6 weeks of pregnancy are administered a single course of antenatal corticosteroids (betamethasone or dexamethasone, 2 × 12 mg intramuscularly with a 24-hour interval) if classified at risk of preterm birth. This is based on several factors, such as medical history, preterm contractions, cervical ripening, fibronectin levels, premature rupture of membranes, and vaginal bleeding. By the 1990 s, the antenatal use of corticosteroids for preterm birth risk was widely adopted, with betamethasone and dexamethasone being the most widely used agents today. Although current evidence suggests that there is no significant difference in efficacy between these two drugs, additional research is warranted [4].

In addition to the context of preterm birth, antenatal corticosteroids have also been utilised since the 1980 s for pregnancies affected by congenital adrenal hyperplasia [1]. Congenital adrenal hyperplasia refers to a group of autosomal recessive conditions affecting adrenal steroidogenesis. The most common cause is 21-hydroxylase deficiency due to mutations in the *CYP21A2* gene. This disrupts hormone levels, favouring the production of androgens over cortisol and aldosterone. The two main types of congenital adrenal hyperplasia are classic and non-classic. Classic congenital adrenal hyperplasia is rarer with the incidence in Western countries estimated to be 1/15,000–20,000 births [5]. Additionally, it is more severe, presents earlier in life and can result in virilisation (masculinisation) of the external genitalia in female fetuses. Postnatally, individuals with classic forms of congenital adrenal hyperplasia require lifelong hormone replacement therapy. Surgical interventions may also be indicated including genital reconstructive surgery, though this is not always needed. Consistent prenatal dexamethasone treatment, from before gestational week ~ 6–8 (before external genitalia differentiation) until delivery, has been shown to effectively prevent/reduce virilisation in pregnancies at risk of having a congenital adrenal hyperplasia-affected fetus. This works via suppressing fetal androgen production and thus reduces the need for later reconstructive surgery [6]. Prenatal dexamethasone treatment is stopped in males or unaffected females, when sex or congenital adrenal hyperplasia status is determined.

Since fetal sex and congenital adrenal hyperplasia status were traditionally determined by chorionic villus sampling (~ 10–12 weeks) or amniocentesis (~ 15–20 weeks), approximately seven out of eight fetuses (males and non-congenital adrenal hyperplasia affected females) were exposed to ~ 4-12weeks of unnecessary treatment [7, 8]. Miller [9] estimates that the fetus receives glucocorticoid concentrations around 60 times higher than physiological cortisol levels when utilising the dosage regimen 20 µg/kg/day. Additionally, concerns regarding long-term outcomes following first-trimester dexamethasone, including changes in behaviour, cognition and endocrine systems, have made this treatment controversial. For example, Trautman et al. [10] reported developmental and behavioural delays in children exposed prenatally, while Hirvikoski et al. [11] reported deficits in cognitive scores and increased social anxiety in those between seven and 17 years of age. More recently, Riveline et al. [12] reported altered insulin and glucagon responses following metabolic challenges in adults who were exposed prenatally from gestational weeks six to 18. Given these concerns, the Endocrine Society [13] currently advise that antenatal corticosteroid treatment for congenital adrenal hyperplasia should be regarded as experimental, and that research utilise modern techniques, including using cell-free fetal DNA isolated from the circulation of pregnant individuals, to exclude male fetuses from inadvertent treatment [6]. Tardy-Guidollet [6] recommend SRY testing before the sixth week of gestation but after the 4.5 week to reduce false-negatives but still ensure sex is determined before potential glucocorticoid administration. Despite this, unnecessary treatment of females without congenital adrenal hyperplasia would be unavoidable. However, in 2014, testing on cell-free fetal DNA was conducted to detect *CYP21A2* mutations, detecting fetal congenital adrenal hyperplasia status as early as 5 weeks and 6 days gestation [14]. This approach may reduce unnecessary dexamethasone exposure by allowing earlier identification of unaffected pregnancies. Currently, prenatal dexamethasone is not a standard practice in many countries, including the United Kingdom, the United States and Sweden. Hanna Nowotny [8] has made an inventory of prenatal dexamethasone practices for congenital adrenal hyperplasia in Europe.

Males have higher rates of preterm birth, as well poorer outcomes compared to females in those preterm born [15], including higher rates of intraventricular haemorrhage [16], higher rates of respiratory morbidity and mortality [17] and poorer neurodevelopmental outcomes [18]. However, it remains unclear whether antenatal corticosteroid treatment results in sexually-dimorphic outcomes. In contrast, the role of fetal sex has been central to many studies of antenatal corticosteroids in congenital adrenal hyperplasia, as treatment decisions are directly influenced by fetal sex. As a result, research on congenital adrenal hyperplasia has more consistently focused on the implications of sexual dimorphisms.

**Fig. 1:**
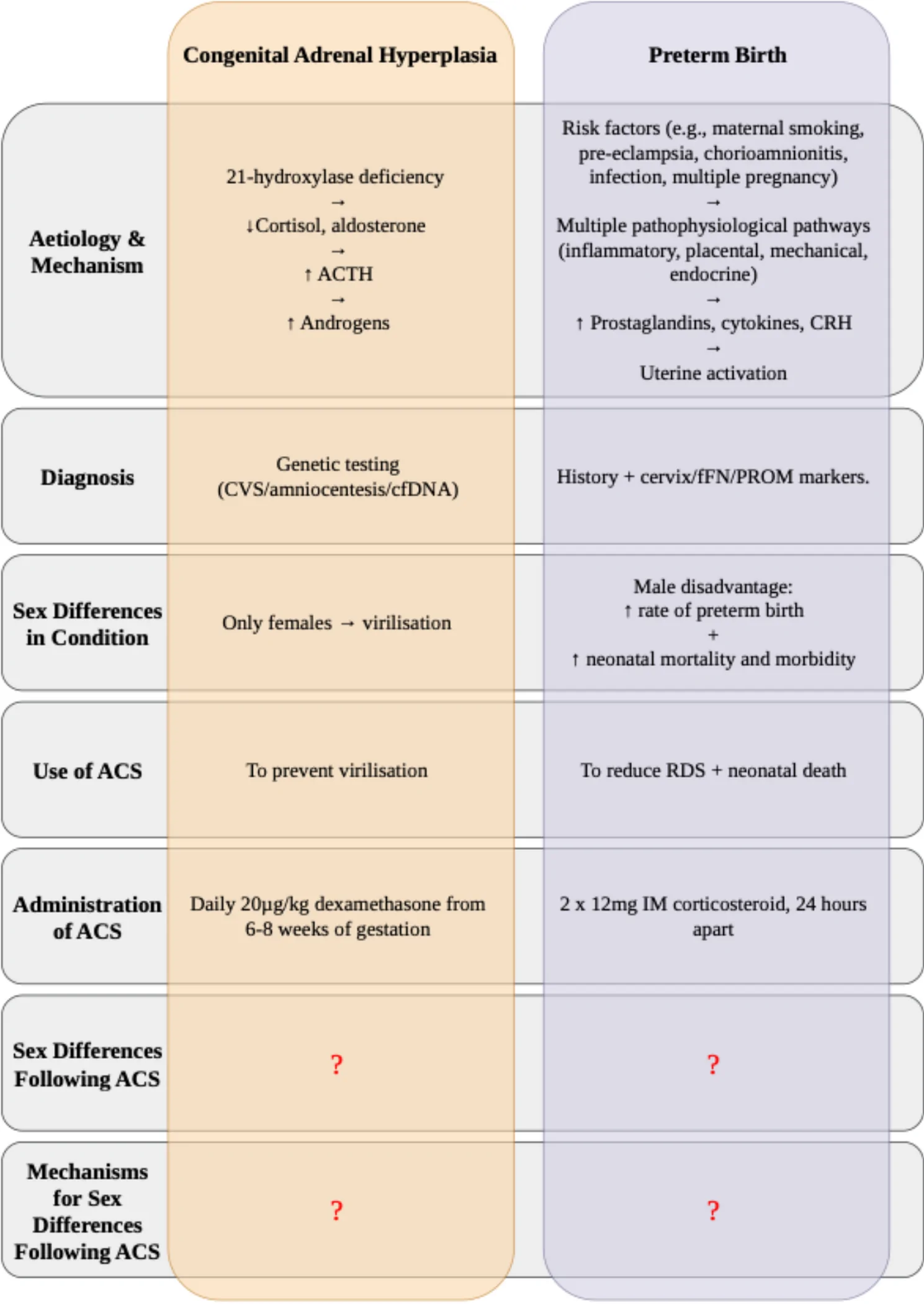
**Overview of known and unknown aspects of congenital adrenal hyperplasia and preterm birth risk.** The figure compares what is known and unknown about congenital adrenal hyperplasia and preterm birth risk across key domains, with a focus on antenatal corticosteroids. Red question marks denote knowledge gaps and highlight the focus of this review. Antenatal dexamethasone for congenital adrenal hyperplasia was historically administered daily before 6–8 weeks of gestation until delivery (or until fetus was found to be unaffected or male), first proposed in 1984 [1] however, this is not currently recommended clinical practice [2].ACS, antenatal corticosteroids; cfDNA, cell-free fetal DNA; CVS, chorionic villus sampling; fFN, fetal fibronectin; IM, intramuscular; PROM, premature rupture of membranes; RDS, respiratory distress syndrome.

The aim of this review was to evaluate the evidence of sexually-dimorphic offspring outcomes following antenatal corticosteroids for congenital adrenal hyperplasia and preterm birth risk. It also explored the potential mechanisms underlying sexually-dimorphic outcomes. A search of the MEDLINE database was conducted, with a focus on clinical trials that compared sex-specific outcomes in response to antenatal corticosteroids for each condition. Understanding potential differences in outcomes between the sexes and with different conditions is crucial for optimising antenatal corticosteroid use and improving outcomes for both sexes. Figure 1 outlines the known and unknown aspects of antenatal corticosteroids in the context of congenital adrenal hyperplasia and preterm birth risk.

## Methods

The terms “sex”, “male”, and “female” will be used to refer to chromosomal makeup/assigned sex at birth, without making assumptions about gender identity. In addition, the term ‘pregnant people’ will be utilised to include all who may be pregnant. For consistency, gestational age at birth was categorised according to the World Health Organisation (WHO) definitions: preterm < 37 weeks, extremely preterm < 28 weeks, very early preterm between 28 and 32 weeks and moderate-to-late preterm between 32 and 37 weeks [19].

**Fig. 2:**
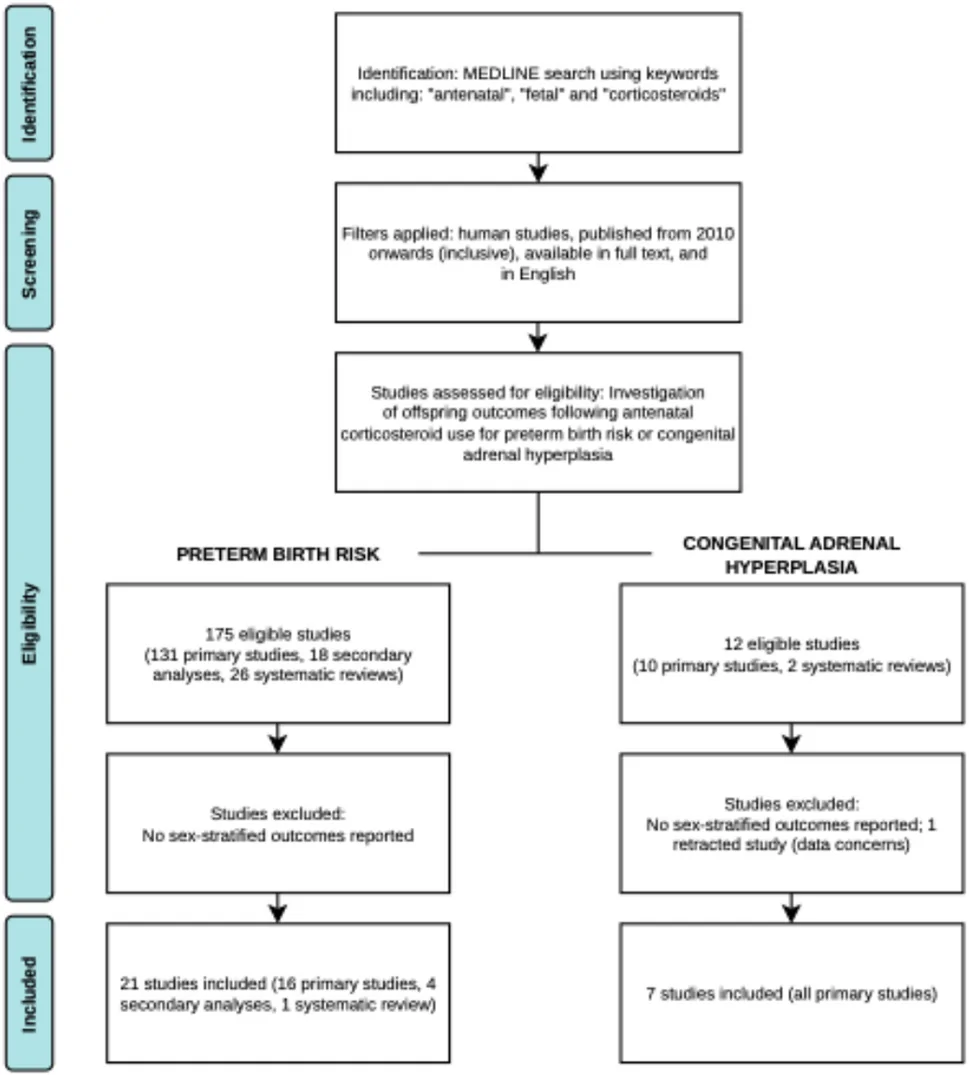
**Flow diagram of study selection process.** The figure shows the study selection process for both the congenital adrenal hyperplasia and preterm birth studies. Studies were identified through a MEDLINE search (February 2025). Following screening, studies investigating offspring outcomes following antenatal corticosteroid use for preterm birth risk or congenital adrenal hyperplasia were identified. Studies were then assessed for whether outcomes were stratified by sex, studies were excluded if sex-stratified outcomes were not reported. One congenital adrenal hyperplasia study was additionally excluded due to retraction.

A comprehensive MEDLINE (accessed via PubMed) search was conducted in February 2025, using keywords, including, ‘prenatal’, ‘antenatal’, ‘fetal’, ‘neonatal’, ‘corticosteroids’, ‘glucocorticoids’, ‘betamethasone’, ‘dexamethasone’, and ‘prednisone’. Specific search terms were included for congenital adrenal hyperplasia due to its rarity; preterm birth studies were identified through corticosteroid-related keywords. Searches were limited to human studies published from 2010 onwards (inclusive), available in full text and in English. No additional databases were searched (Fig. 2). 

Study selection was performed in two stages by a single reviewer. In the first stage, studies were assessed for eligibility based on investigation of offspring outcomes, at any age, following antenatal corticosteroid exposure for either congenital adrenal hyperplasia or preterm birth risk. All types of outcomes were considered, including but not limited to neurodevelopmental, respiratory, anthropometric, and metabolic outcomes. This broad inclusion approach was chosen to capture the full scope of potential sexually dimorphic effects. Studies including offspring at any age were eligible, acknowledging that differences in neonatal care over time may contribute to heterogeneity between studies. Studies including term-born infants who were treated for suspected preterm birth risk were retained. Studies restricted to parental or placental outcomes were excluded. Only full-text articles were included.

In the second stage, eligible studies were further assessed for inclusion based on reporting of offspring outcomes stratified by sex. Only studies meeting this criterion were included in the final analysis, irrespective of whether sex-specific differences were identified. Risk of bias and publication bias were not formally assessed, study quality was considered qualitatively, as described in the critical appraisal section. A narrative synthesis of included studies was undertaken. A flow diagram summarising the study selection process is provided (Fig. 2).

## Results

### Study selection and characteristics

Table 1 summarises all the included studies.

**Table 1:** Summary of included studies on antenatal corticosteroid exposure

CAH or preterm birth risk	Study groups	Study (country)	Main aim	Study design	Assessment ages	Gestational age at birth	ACS regime	Main findings	Total size of study population
CAH	DEX Non-CAH vs. Non-DEX Non-CAH	Hirvikoski et al., 2011 [] (Sweden)	Effects of prenatal DEX on gender role behaviour	Cohort pilot study	Range = 7-17yrs, Mean = 10.69yrs	Not reported	DEX 20 µg/kg/day divided into 3 doses (based on pre-pregnancy weight), start 6-7wk	Increased neutral behaviours in DEX non-CAH males than Non-DEX non-CAH males	220
		Karlsson et al., 2018 [] (Sweden)	Effects of prenatal DEX on long-term behaviour and cognitive outcomes	Case-control study	Mean = 20.68yrs	Not reported	DEX, mean treatment length 6.2 ± 2.2wks	No significant behavioural or cognitive outcomes in adults exposed prenatally to DEX	81
		van’t Westeinde et al., 2020 [] (Sweden)	Effects of prenatal DEX on cognitive outcomes in adolescents and adults	Cohort study	Range = 16-26yrs, Mean = 20.41yrs	Not reported	DEX, from 5-9wks, mean treatment length 6wks	Structural brain changes, with accompanying gene methylation changes, in adults exposed prenatally to DEX	62
		Wallensteen et al., 2016 [] (Sweden)	Sex-dimorphic effects of prenatal DEX on brain structure in adults	Cohort study	Range = 7-17yrs, Mean = 10.47yrs	Mean = 39.31wks	DEX 20 µg/kg/day, mean start 6.1wk	Widespread negative cognitive outcomes in DEX non-CAH treated females	100
	DEX CAH vs. Non-DEX CAH	Karlsson et al., 2017 [] (Sweden)	Effects of prenatal DEX on cognitive outcomes in adolescents and adults	Case-control study	Range = 16-33yrs, Mean = 22.31yrs	Not reported	DEX	Impaired executive function in CAH individuals compared with population controls. Poorer general cognitive capacity in null CAH genotype	113
		Messina et al., 2020 [38] (Sweden)	Effects of prenatal DEX on cognitive outcomes in children and adolescents	Cohort study	Range = 7-17yrs, Mean = 11.11yrs	Mean = 39.46wks	DEX, from ~7wk	Normal cognitive outcomes in CAH children and adolescents who were diagnosed early and treated with hydrocortisone	95
	Mixed comparisons	Meyer-Bahlburg et al., 2012 [37] (U.S.A)	Effects of prenatal DEX on cognitive outcomes in children	Cohort study	Range = 5-12yrs, Mean = 8.11yrs	Not reported	DEX	Negative cognitive function in CAH females with long-term DEX exposure, but not in short-term DEX exposure	140
Preterm birth risk	ACS-exposed vs. non-exposed (term-born)	Alexander et al., 2016 [26] (Germany)	Effects of ACS on intelligence in term-born children	Cross-sectional study	Range = 6–11 yrs, Mean = 8.4, SD = 1.3	Term (37-41wks)	BETA or DEX, 2 × 12 mg with 24 h interval	Lower IQ in children born to those at risk of preterm delivery, irrespective of ACS	222
	ACS-exposed vs. non-exposed (term-born)	Alexander et al., 2012 [27] (Germany)	Effects of ACS on endocrine stress reactivity in term-born children	Cross-sectional study	Range = 6–11 yrs, Mean = 8.4, SD = 1.3	Term (37-41wks)	BETA or DEX, 2 × 12 mg with 24 h interval	Increase cortisol reactivity in ACS exposed term-born children	209
	BETA-exposed vs. non-exposed (preterm twins)	Braun et al., 2021 [31] (Germany)	Long-term effects of ACS on growth in preterm twin children	Cohort follow-up study	At birth, 21-24mo, 60-64mo	Preterm (25 + 0–36 + 6wks)	BETA, ≤ 16 mg, ≤ 24 mg or > 24 mg	Long-term growth changes, including ponderal mass index and BMI, in ACS exposed twins	99 twin pairs (198 individuals)
	BETA-exposed vs. non-exposed, stratified by gestational age	Braun et al., 2015 [40] (Germany)	Effects of BETA on fetal and neonatal outcomes in preterm and term born children	Cohort study	At birth	Very Early Preterm, Early Preterm, Late Preterm, Term, Postterm^a^	BETA, ≤ 16 mg, ≤ 24 mg or > 24 mg	Reduced fetal weight gain in dose-dependent manner, in BETA exposed children	44,039
	BETA-exposed vs. non-exposed (twins), stratified by gestational age	Braun et al., 2016 [30] (Germany)	Effects of BETA on fetal and neonatal outcomes in preterm and term born twin children	Cohort study	At birth	Early Preterm, Late Preterm, Term	BETA, ≤ 16 mg, ≤ 24 mg or > 24 mg	Reduced fetal growth in dose-dependent manner, in BETA exposed twin children	1922 twin pairs (3844 individuals)
	BETA vs. placebo	Gyamfi-Bannerman et al., 2016 [32] (U.S.A)	Effects of BETA on fetal and neonatal outcomes in pregnancies at risk of late preterm delivery	Multi-centre RCT	At birth, 28d	Late Preterm (34 + 0–36 + 6wks)	BETA, 2 × 12 mg with 24 h interval	BETA reduced respiratory complications in pregnancies at risk of late preterm delivery	2,827
	ACS-exposed vs. non-exposed (term-born)	Hjalmarson & Sandberg, 2011 [46] (Sweden)	Effects of ACS on lung function in term born infants	Case-control study	Infant	Term (37-42wks)	BETA, 1, 2 or 3 × 12 mg	No long-term lung structure or function abnormalities in ACS exposed term born infants	72
	BETA (12 h interval) vs. BETA (24 h interval)	Kashanian et al., 2018 [47] (Iran)	Comparison between two doses of BETA with 12 h vs. 24 h intervals on prevention of RDS	RCT	At birth	Preterm	BETA, 2 × 12 mg with 12 h interval or 2 × 12 mg with 24 h interval	BETA with 12 h interval may be an effective alternative ACS regime for preterm birth risk	201
	ACS-exposed vs. non-exposed	Laugesen et al., 2025 [64] (Denmark)	Effects of ACS on mental disorders	Cohort study	Median = 9yrs, IQR = 7–14	Preterm	Mostly BETA, 2 × 12 mg with 24 h interval	Increased risk of mental disorders in ACS exposed adolescents	31,518
	ACS-exposed vs. non-exposed (extremely preterm)	Lee et al., 2022 [41] (UK)	Effects of ACS on fetal and neonatal outcomes in extremely prematurely born infants	Cohort study	At birth	Extremely Preterm (< 28wks)	Any	ACS had a greater beneficial effect in females (compared to males) in extremely prematurely born infants	11,714
	BETA-exposed vs. non-exposed	Ramos-Navarro et al., 2022 [42] (Spain)	Effects of ACS and sex on mortality and morbidity in preterm infants	Cohort study	During hospital stay, During first 24mo	< 32wks	BETA, 2 × 12 mg with 24 h interval	ACS had a greater beneficial effect in males (compared to females) in infants born < 29wks	710
	BETA-exposed vs. non-exposed	Rodriguez et al., 2019 [73] (Finland)	Effects of ACS on size at birth	Cohort study	At birth	Very Preterm, Preterm, Near Preterm, Term, Postterm^b^	BETA, 2 × 12 mg with 24 h interval	Reduced infant birth size in ACS exposed infants born preterm, near term or at term	278,508
	BETA-exposed vs. non-exposed	Stark et al., 2011a [28] (Australia)	Effects of ACS and sex on preterm placental pro-oxidant-antioxidant balance	Cohort study	Immediate post-delivery (placenta samples)	24-31wks	BETA, 1 × 11.4 mg with 24 h interval or 2 × 11.4 mg with 24 h interval	Sex-specific differences in placental oxidative stress in ACS exposed infants	60
	BETA-exposed vs. non-exposed	Stark et al., 2011b [29] (Australia)	Effects of BETA and sex on vasoconstrictors and the preterm microvasculature	Cohort study	At birth	24-28wks, 29-32wks	BETA, 1 × 11.4 mg with 24 h interval or 2 × 11.4 mg with 24 h interval	Sex-specific differences in mediators of vascular tone in ACS exposed infants	60
	Late BETA-exposed vs. non-exposed, stratified by singleton/twin status	Vieira et al., 2022 [45] (U.S.A)	Effects of late ACS on respiratory health and glycaemic control in singletons and twins	Cohort study	First 75 h post-birth	Late Preterm (34 + 0–36 + 6wks)	BETA, 2 × 12 mg with 24 h interval	Increased risk of hypoglycaemia in ACS exposed singletons and twins	4,341
	BETA vs. placebo	Walters et al., 2024 [34] (New Zealand)	Cardiovascular outcomes 50yrs after BETA	RCT follow-up	Mean = 49.3 year, SD = 1.0	24-31wks	BETA, 2 × 12 mg with 24 h interval or 2 × 24 mg with 24 h interval	No significant cardiovascular outcomes in ACS exposed adults	424
	BETA vs. placebo	Battarbee et al., 2019 [33] (U.S.A)	Effects of sex in BETA exposed individuals born late preterm	Secondary analysis	At birth	Late Preterm (34 + 0–36 + 6wks)	BETA	Worse outcomes in males following BETA in late preterm born infants	2,331
	Single vs. multiple courses of ACS	Ninan et al., 2024 [74] (20 different countries)	Effects of single vs. multiple courses of ACS and sex on perinatal outcomes	Secondary analysis	At birth	Preterm	Mostly BETA (some DEX, 1 unknown)	No significant effect of sex on perinatal outcomes in ACS exposed individuals	2,304
	BETA vs. placebo	St Clair et al., 2024 [35] (New Zealand)	Reproductive outcomes 50yrs after BETA	Secondary analysis	Mean = 49.3 year, SD = 1.0	Preterm	BETA, 2 × 12 mg with 24 h interval or 2 × 24 mg with 24 h interval	No significant reproductive outcomes in ACS exposed adults	415
	BETA vs. placebo	Walters et al., 2023 [36] (New Zealand)	Secondary analysis of AST	Secondary analysis	At birth	Preterm	BETA, 2 × 12 mg with 24 h interval or 2 × 24 mg with 24 h interval	Reduced RDS in ACS exposed infants born between 24-37wks gestation, with greater effect in males compared to females	1,218

This table summarises the characteristics of all included studies, including main aim, study design, assessment ages, gestational age at birth, antenatal corticosteroid regimen, main findings, and total size of study population

ACS, antenatal corticosteroids; BETA, betamethasone; CAH, congenital adrenal hyperplasia; DEX, dexamethasone; RCT, randomised control trial; SD, standard deviation

For congenital adrenal hyperplasia, 11 studies met the initial eligibility criteria (nine primary studies and two systematic reviews). Following second-stage screening, seven studies were included in the final analysis (Table 2); four were excluded due to absence of sex-stratified outcome reporting. All seven of the included studied reported sex-specific differences in offspring outcomes.

Six of the seven included studies (Table 2) were conducted by the same research group in Sweden and included only Swedish participants. One of these studies was a pilot study [20]. Known instances of overlapping participants include Van’t Westeinde et al. [21], who selected participants from the PREDEX study, which includes three of the selected studies [22–24], and Wallensteen et al. [22], who included participants who were also in Hirvikoski et al. [20]. Due to the rarity of both the disease and its treatment, only 77 pregnancies in Sweden were treated with prenatal dexamethasone for risk of congenital adrenal hyperplasia between 1984 and 2010, with many individuals likely included in multiple studies. The characteristics of all seven studies are reported in both Tables 1 and 2.

**Table 2:** Studies investigating sex-specific outcomes following antenatal corticosteroid therapy for congenital adrenal hyperplasia

Study groups	Study (country)	Assessment ages	Key considerations	Summary of sex-specific outcomes	Sample size of interest
DEX Non-CAH vs. Non-DEX Non-CAH	Hirvikoski et al., 2011 [20] (Sweden)	Range = 7-17yrs, Mean = 10.62yrs	CAH-affected children could not be analysed on own due to small sample size	↑Variance for behaviours - Non-CAH (+/- CAH) M	60
			KI-GRB inventory was new, disallowing comparison of results from other studies	↑Neutral behaviours - Non-CAH (+/- CAH) M	
			Small sample size		
	Karlsson et al., 2018 [24] (Sweden)	Mean = 20.68yrs	Small sample size	Interaction between DEX and sex for vocabulary	81
			No correction for multiple analyses (stated to prevent missing effects that are clinically relevant)		
	van’t Westeinde et al., 2020 [21] (Sweden)	Range = 16-26yrs, Mean = 20.41yrs	DNA methylation data only on proportion of individuals (~ 50% total population)	↑AD - Non-CAH M	62
			Small sample size	↓FA - Non-CAH F	
	Wallensteen et al., 2016 [22] (Sweden)	Range = 7-17yrs, Mean = 10.47yrs	Not all individuals completed questionnaire for scholastic performance	↓ General intelligence - Non-CAH F	100
			Small sample size	↓ Forward span board test - Non-CAH F	
DEX CAH vs. Non-DEX CAH	Karlsson et al., 2017 [23] (Sweden)	Range = 16-33yrs, Mean = 22.31yrs	Stroop test scores were converted using American norms as no Swedish norms available	↓ General intelligence & Executive function - CAH F	113
			No correction for multiple analyses (stated to prevent missing effects that are clinically relevant)	↓Long-term memory - CAH M + F	
			Small sample size, particularly within DEX CAH group (5 male + 4 female)		
	Messina et al., 2020 [38] (Sweden)	Range = 7-17yrs, Mean = 11.45yrs	Stroop + NEPSY test used American norms/manual as no Swedish norms/manual available	↓Vocabulary - CAH F	32
			Study groups were not blinded to psychologists, but were not told about type of DEX or CAH phenotype		
			No correction for multiple analyses (stated to prevent missing effects that are clinically relevant)		
			Small sample size		
Mixed comparisons	Meyer-Bahlburg et al., 2012 [37] (U.S.A)	Range = 5-12yrs, Mean = 8.11yrs	2 individuals start + ending dates of DEX not available	↓Faces & Places scores (visual memory) - Non-CAH F	140
			Due to costs, only proportion of DEX individuals were included	Interaction between DEX and CAH for sequential & mental processing - F	
			No correction for multiple analyses (stated to prevent missing effects that are clinically relevant)	↑Number Recall & Word Order - Non-CAH/CAH M	
			Small sample size		

This table shows the characteristics of the included studies, with a summary of the sex-specific outcomes found. The sample sizes refer specifically to the population relevant to this review and may differ from the total sample size of each study. Assessment age is reported based on the relevant population of interest

AD, axial diffusivity; CAH, congenital adrenal hyperplasia; DEX, dexamethasone; FA, fractional anisotropy; F, female; KI-GRB, Karolinska Inventory of Gender Role Behaviour; M, male; NEPSY, NEuroPSYchological assessment

For preterm birth risk, 175 studies met the initial eligibility criteria (131 primary studies, 18 secondary analysis studies and 26 systematic reviews). Of these, 21 studies (16 primary studies, four secondary analysis studies and one systematic review) met the inclusion criterion of sex-stratified outcome reporting and were included in the final analysis (Table 3) [25]. Among these, 14 studies reported sex-specific differences in outcomes, while seven found no significant sex-based differences.

Three systematic reviews had planned subgroup analyses of outcomes and sex but could not achieve these because of a lack of data. One secondary analysis paper had no stratification by sex despite mentioning sex as a factor influencing neonatal respiratory morbidity. Many of the included studies had overlapping cohorts/datasets. Three pairs of primary studies utilised the same dataset: [26, 27] for births between 1997 and 2003 from Dresden Neustadt Hospital; [28, 29] for births from John Hunter Hospital; and [30, 31] for twins born between 1993 and 2011 at Charité University Hospital. Both the primary Antenatal Late Preterm Steroids (ALPS) study [32] and a secondary analysis of the ALPS study [33] were included. A 50-year follow-up of the Auckland Steroid Trial (AST) [34], a secondary analysis of the 50-year follow-up of the AST [35] and a secondary analysis of the original AST [36] were included, but the original AST was not included as it did not lie within the eligible study criteria dates. The characteristics of the 16 primary studies and the four secondary analysis studies are presented in both Tables 1 and 3. 

### Outcomes for children at risk of congenital adrenal hyperplasia treated prenatally with dexamethasone

Overall, the findings revealed sex-specific outcomes following prenatal dexamethasone treatment for congenital adrenal hyperplasia, with more consistently negative findings in females and mixed outcomes in males.

#### Non-congenital adrenal hyperplasia affected

In the non-congenital adrenal hyperplasia affected population, prenatal dexamethasone treatment was found to be associated with more consistently negative outcomes for females and mixed outcomes in males compared to the reference (untreated) group, including changes in cognitive performance, brain structure and behaviour.

Wallensteen et al. [22] reported that non-congenital adrenal hyperplasia dexamethasone females had significantly lower cognitive scores than the reference group did on tests of general intelligence (digit span, vocabulary, block design) and visuospatial working memory (forward span board). Notably, the fourth general intelligence test (coding) had a near-significant interaction effect (*p* = 0.068). However, the authors “observed no effects on long-term memory, handedness, speed of processing, nor self-perceived or parentally reported scholastic performance”. Meyer-Bahlburg et al. [37], an American study, reported significantly decreased performance on the Faces & Places test in non-congenital adrenal hyperplasia dexamethasone females, a visual memory test. However, Meyer Bahlburg et al. [37] conclude overall that their findings do not replicate the findings of adverse working memory outcomes following short-term dexamethasone treatment in non-congenital adrenal hyperplasia females. Additionally, the authors reported greater sequential processing in non-congenital adrenal hyperplasia dexamethasone males than in the reference group, with significantly higher scores for both number recall and word order tests. Similar findings were observed in males with congenital adrenal hyperplasia. The authors noted that their findings were not definitive and recommended replication in larger samples.

**Table 3:** Studies investigating sex-specific outcomes following antenatal corticosteroid therapy for preterm birth risk

Study (country)	Study design	Assessment ages	Gestational ages	Key considerations	Summary of Sex-specific outcomes	Sample size analysed
Alexander et al., 2016 [26] (Germany)	Cross-sectional study	Range = 6–11 yrs, Mean = 8.4, SD = 1.3	Term (37-41wks)	Those relating to observational studies	None	222
				No data on whether singletons and/or twins		
Alexander et al., 2012 [27] (Germany)	Cross-sectional study	Range = 6–11 yrs, Mean = 8.4, SD = 1.3	Term (37-41wks)	Those relating to observational studies	↑HPA axis activity - preterm F	209
				No data on whether singletons and/or twins		
Braun et al., 2021 [31](Germany)	Cohort follow-up study	At birth, 21-24mo, 60-64mo	Preterm (25 + 0–36 + 6wks)	Those relating to observational studies	Head circumference - preterm M > preterm F	99 twin pairs (198 individuals)
				Web-based calculator used for growth percentiles based on singleton data not twins	Change in growth trajectories - preterm F	
				Heterogenous ACS regimes		
				No reference (untreated) group		
Braun et al., 2015 [40] (Germany)	Cohort study	At birth	Very Early Preterm, Early Preterm, Late Preterm, Term, Postterm^a^	Those relating to observational studies	↓BW - M (34 + 0–39 + 6wks), F (> 31 + 0wks)	44,039
				Heterogenous ACS regimes	↓Head circumference - M (31 + 0–36 + 6wks), F (> 34 + 0wks)	
				Reference group limitation	↓BL - M (34 + 0wks), F (> 37 + 0wks)	
					↓APGAR - M (34 + 0–39 + 6wks), F (> 40 + 0wks)	
					↑APGAR - M (31 + 0–33 + 6 wks)	
					↑Mortality - M (< 37wks), F (< 37wks and > 37wks)	
Braun et al., 2016 [30] (Germany)	Cohort study	At birth	Early Preterm, Late Preterm, Term	Those relating to observational studies	↓BW & Head circumference - term F twins	1,922 twin pairs (3,844 individuals)
				Heterogenous ACS regimes	↓BW - late preterm M < F	
				Reference group limitation	↓BW & BL - F in mixed twin pairs	
Gyamfi-Bannerman et al., 2016 [32] (U.S.A)	Multi-centre RCT	At birth, 28d	Late Preterm (34 + 0–36 + 6wks)	~ 60% compliance to treatment/placebo (can lower power)	None	2,827
Hjalmarson & Sandberg, 2011 [46] (Sweden)	Case-control study	Infant	Term (37-42wks)	Those relating to observational studies	None	72
				Unclear whether SGA were included or not Reference group were not matched for BW + GA + sex (although mean GA was no different between groups)		
				Small sample size of treatment group		
				Heterogenous ACS regimes		
Kashanian et al., 2018 [47] (Iran)	RCT	At birth	Preterm	Unclear whether SGA were included or not Statistically significant difference in GA between 12 h interval and 24 h interval groups, was adjusted for but limited due to small sample sizes within strata	None	201
				No control (untreated) group		
Laugesen et al., 2025 [64] (Denmark)	Cohort study	Median = 9yrs, IQR = 7–14	Preterm	Those relating to observational studies	None	31,518
				Some incomplete parental information		
				Heterogenous ACS regimes		
Lee et al., 2022 [41] (UK)	Cohort study	At birth	Extremely Preterm (< 28wks)	Those relating to observational studies	↓Mortality - M, F (M < F)	11,714
				No data on completeness/timing/regime/type of ACS	Mortality rates M > F	
				No data on whether singletons and/or twins		
				No data on parental baseline characteristics		
Ramos-Navarro et al., 2022 [42] (Spain)	Cohort study	During hospital stay, During first 24mo	< 32wks	Those relating to observational studies	↓Mortality - M (2.37-fold)	710
				No data on whether singletons and/or twins	↓Survival level without BPD grades 2–3 - M (26-32wks)	
				Follow-up data only completed in subset (*n* = 337) Included those IUGR, but unclear whether any difference between groups for IUGR, and BW not treated as a confounding factor		
Rodriguez et al., 2019 [73] (Finland)	Cohort study	At birth	Very Preterm, Preterm, Near Preterm, Term, Postterm^b^	Those relating to observational studies	↓APGAR - M (38-41wks), F (35-37wks)	278,508
				Missing values in FMBR	↑APGAR - F (> 38wks)	
Stark et al., 2011a [28] (Australia)	Cohort study	Immediate post-delivery (placenta samples)	24-31wks	Those relating to observational studies	↑> Grade 2 IVH & Respiratory help - M (born < 72 h after ACS)	60
				No data on whether singletons and/or twins	Time between ACS & Delivery - M < F (born > 72 h after ACS)	
				No reference (untreated) group		
				Small sample size		
Stark et al., 2011b [29] (Australia)	Cohort study	At birth	24-28wks, 29-32wks	Those relating to observational studies	Normetanephrine in urine - M < F (born < 72 h after ACS)	60
				No reference (untreated) group	Umbilical Endothelin-1 - M < F (born < 72 h after ACS)	
				Small sample size		
Vieira et al., 2022 [45] (U.S.A)	Cohort study	First 75 h post-birth	Late Preterm (34 + 0–36 + 6wks)	Those relating to observational studies	Respiratory complications - M > F (singletons)	4,341
				Small sample size of completed ACS course in twins (~ 40%), may not be enough to detect associations	Rates of hypoglycaemia - M > F (twins)	
Walters et al., 2024 [34] (New Zealand)	RCT follow-up	Mean = 49.3 year (SD = 1.0)	24-31wks	Low follow-up rate (46% of presumed surviving offspring)	Interaction between ACS and sex for rates of overweight	424
				Rate for major adverse cardiovascular events lower than expected, reducing power		
				Large number of participants lost to follow-up, possibly cause survival bias		
Battarbee et al., 2019 [33] (U.S.A)	Secondary analysis	At birth	Late Preterm (34 + 0–36 + 6wks)	Limited by data collection from primary trial, including lack of specific reason for NICU admissions	Severe neonatal morbidity (M > F)	2,331
				Unclear whether SGA included (but likely) Considerations from the primary trial (Gyamfi-Bannerman et al., 2016) (32)		
Ninan et al., 2024 [74] (20 different countries)	Secondary analysis	At birth	Preterm	Possible covariates (e.g., IVH, necrotising enterocolitis and cystic periventricular leukomalacia) not accounted for due to low rates	None	2,304
St Clair et al., 2024 [35] (New Zealand)	Secondary analysis	Mean = 49.3 year (SD = 1.0)	Preterm	Self-reported data is subject to recall bias	↓Age at first child - M	415
				No adjustments made for multiple comparisons, increasing risk of type 1 errors		
				Considerations from the primary trial (Walters et al., 2024) (34)		
				Small sample size for some strata		
Walters et al., 2023 [36] (New Zealand)	Secondary analysis	At birth	Preterm	Heterogenous ACS regimes	↓RDS - M, F (M > F)	1,218
				Conducted 50 years ago - may limit generalisability to present day		
				Methods used to measure GA, and other parameters were less accurate than modern techniques		
				Use of cortisone as placebo, which is minimally active		
				Small sample size for some strata		

ACS, antenatal corticosteroids; APGAR, Appearance, Pulse, Grimace, Activity and Respiration score; BL, birth length; BPD, bronchopulmonary dysplasia; BW, birthweight; F, female; FMBR, Finnish Medical Birth Register, GA, gestational age; HPA, hypothalamic–pituitary–adrenal axis; IUGR, intrauterine growth restriction; IVH, intraventricular haemorrhage; M, male; M1/M0, moment ratio (measurement of gas-mixing efficiency); NICU, neonatal intensive care unit; RCT, randomised control trial; RDS, respiratory distress syndrome; SGA, small-for-gestational-age

This table shows the characteristics of the included studies, with a summary of the sex-specific outcomes found. “Gestational age” was not classified by NICE categorisation, but by specification in the respective study

^a^ Very early preterm (group 1 = 25 + 0–27 + 6 weeks, group 2 = 28 + 0–30 + 6 weeks), early preterm (31 + 0–33 + 6 weeks), late preterm (34 + 0–36 + 6 weeks), term (37 + 0–39 + 6 weeks), and postterm (≥ 40 weeks)

^b^ Very preterm (24–29 weeks), preterm (30–34 weeks), near preterm (35–37 weeks), term (38–41 weeks) and postterm (≥ 42 weeks)

Meyer Bahlburg et al. [37] reported no significant differences between non-congenital adrenal hyperplasia females with and without prenatal dexamethasone for number recall, which contrasts with the findings of Wallensteen et al. [22] for digit span. Both tests examine the ability to recall a series of heard numbers (a measure of short-term and working memory), but the digit span test is typically conducted with both forward and backwards components, whereas the number recall test is only forward.

Hirvikoski et al. [20] reported a significantly greater level of neutral behaviours in non-congenital adrenal hyperplasia dexamethasone males and a significantly different variance for all behaviours (masculine, feminine and neutral) than in the reference group. This was observed when also including congenital adrenal hyperplasia affected males. No significant changes were found in females.

Karlsson et al. [24] noted a significant interaction between prenatal dexamethasone and sex for vocabulary within non-congenital adrenal hyperplasia populations, where vocabulary was increased in males, but decreased in females.

#### Congenital adrenal hyperplasia affected

Like in the non-congenital adrenal hyperplasia population, prenatal dexamethasone treatment among congenital adrenal hyperplasia-affected individuals was found to be associated with negative cognitive outcomes for females and mixed cognitive outcomes for males. Interpretation of sex differences in this population is challenging, as males and females are exposed to different durations of prenatal dexamethasone. In clinical practice, treatment is continued for longer in affected females than in males, limiting direct comparability between sexes. Notably, much of the evidence is derived from within-sex comparisons (i.e., treated vs. untreated individuals within males and females separately), which partially mitigates this limitation. Within this context, a greater impact of prenatal dexamethasone was found in females, consistent with the longer duration of exposure.

Three studies (Table 2) reported that females with congenital adrenal hyperplasia who had prenatal exposure to dexamethasone had decreased general intelligence, executive functions and long-term memory compared to those without. Additionally, long-term memory was also lower in congenital adrenal hyperplasia males who had been treated with prenatal dexamethasone compared to those without. However, the effect size in males was smaller than that in females [23].

A significant interaction between prenatal dexamethasone and congenital adrenal hyperplasia for sequential and mental processing outcomes in females was also found, suggesting that cognitive aspects might be sensitive to long-term prenatal dexamethasone treatment (Table 2). There was no such interaction observed in males [38].

### Preterm birth risk outcomes

Overall, these studies provide a useful starting point for the investigation into sexually-dimorphic effects following antenatal corticosteroids for preterm birth risk. The primary and secondary studies (Table 3) were analysed separately from the systematic review/meta-analysis due to differences in methodology, risk of double-counting studies (although no overlap was found), and to aid in the interpretation and critical appraisal of evidence quality.

#### Primary & secondary studies

16 primary and four secondary studies were included (Table 3), with sex-specific antenatal corticosteroid-related outcomes reported in 14 (14/20).

Sex differences were analysed in various ways: some compared each sex to the respective control/reference groups, whereas others directly compared the sexes following antenatal corticosteroid exposure. Since males have a higher baseline risk for mortality and morbidity [39], known as the “male disadvantage”, reporting better outcomes for females following antenatal corticosteroids could be misleading as they may reflect a true sex difference in efficacy, baseline risk variations, or both.

When comparing sexes following antenatal corticosteroids, more studies reported worse outcomes in males, including higher mortality, higher morbidity, shorter intervals between antenatal corticosteroids and delivery, and lower urine normetanephrine and umbilical endothelin-1 (indicating lower levels of vasoconstrictors) (Table 3). The only outcomes where males fared better were larger head circumference percentiles at 21–24 months, and no significant differences in growth trajectories (which was seen in females) [31].

When comparing antenatal corticosteroid outcomes to those of control/reference groups, there were conflicting data on the effect of antenatal corticosteroids on mortality, both in the overall population and between the sexes. In the overall population, mortality following antenatal corticosteroid administration increased in [30, 40], decreased in [41] and did not significantly change in [36]. However, the latter study noted that this may be due to an insufficient sample size. Ramos-Navarro et al. [42] also reported a decrease in mortality, although the study did not perform a statistical test of significance for the overall population.

Concerning sex differences, Ramos-Navarro et al. [42] reported decreased mortality following antenatal corticosteroids only in males, Lee et al. [41] observed decreases in both sexes but to a greater extent in females, and Braun et al. [40] found increased mortality in both sexes among preterm infants but only in females born at term. Walter et al. [36] did not perform a subgroup analysis for mortality and sex following antenatal corticosteroids.

Antenatal corticosteroids were associated with lower anthropometric values in both sexes, with [30, 31] reporting a greater effect in females. When comparing antenatal corticosteroid outcomes to those of control/reference groups, rates of respiratory conditions were also found to primarily decrease in the overall population. However, there is inconclusive evidence for the interaction effect of sex on the rate of respiratory conditions. Ramos-Navarro et al. [42] reported increased rates of type 2–3 bronchopulmonary dysplasia for males born at 26–32 weeks (no differences were found in females), whereas Walters et al. [36] reported lower rates of respiratory distress syndrome in both sexes but to a greater extent in males. Bronchopulmonary dysplasia is a chronic lung condition that can develop in premature infants, most of whom initially present with respiratory distress syndrome.

Five studies examined term-born infants who received antenatal corticosteroids for risk of preterm birth, and reported increased risks compared with control/reference groups, including lower IQ, reduced anthropometric values, and lower Appearance, Pulse, Grimace, Activity and Respiration (APGAR) scores (Table 3). Concerning sex differences in term-born infants, females appeared more vulnerable, with increased mortality rates and reductions in anthropometric measures not observed in males. Additionally, term-born females, following antenatal corticosteroids, were found to have higher hypothalamic-pituitary-adrenal (HPA) axis activity [27], which may be linked to stress-related disorders but may be beneficial for blood pressure regulation.

The Antenatal Late Preterm Steroids primary study [32] reported no significant sex-specific antenatal corticosteroid-related outcomes, whereas the secondary analysis [33] reported higher severe neonatal morbidity in males than females following antenatal corticosteroid treatment, which was not observed in the non-antenatal corticosteroid group. However, the primary study’s borderline p-value (0.06) indicates a trend towards greater efficacy of antenatal corticosteroids in females.

#### Systematic review

The systematic review [25] reported that antenatal corticosteroids reduced respiratory distress syndrome rates, severe intraventricular haemorrhage (grades 3–4) and neonatal mortality. Despite severe intraventricular haemorrhage being significantly reduced in males but not females, no significant sex differences were found for any outcomes with enough data to estimate the p-value for interaction.

The only sex-specific antenatal corticosteroid-related outcome reported was variation in efficacy observed in males, but not females. Betamethasone was associated with a significant reduction in respiratory distress syndrome in males only, and dexamethasone in females only. However, the study’s sensitivity analysis identified a significant difference between antenatal corticosteroid type in only males.

### Critical appraisal of study quality

The quality and consistency of studies investigating antenatal corticosteroid use vary greatly between those focusing on congenital adrenal hyperplasia and those focused on preterm birth risk. These contrasts reflect both the differences in prevalence and the known complications of each condition. Given the narrative nature of this review, no formal risk-of-bias tool was used; instead, study quality was assessed qualitatively using key study characteristics, the main considerations of each study are outlined in Tables 2 and 3.

#### Study design and sample characteristics

All studies investigating congenital adrenal hyperplasia (Table 1) were observational, a preferable choice owing to the rarity of the condition, despite being less robust than randomised control trials. Methodological characteristics, including small sample sizes and assessment ages (~ 13 years), were highly similar. A major contributing factor is the involvement of the same core group of researchers working from a geographically homogenous Swedish cohort in most of the studies. This geographic homogeneity decreases the risk of confounding factors (e.g., variations in healthcare quality/regulations, socioeconomic factors, environmental exposures, and genetic variability), but limits generalisability, particularly as overlap of participants in studies is likely. The participation rates ranged from 61.1% to 85.7% in the treatment groups and from 26.3% to 55.0% in the untreated groups. Two studies suggested that the complexity/length of the study protocol may have contributed to the low rates of untreated participants. Hirvikoski et al. [20] reported no significant differences between families who agreed and those who refused regarding psychosocial problems or the pregnant individual’s attitude toward future treatment or side effects. No other studies mentioned comparing populations who participated versus those who refused. Low participation rates, particularly in the untreated group, raise concerns about selection bias.

In contrast, the preterm birth studies (Table 1) included a wider mix of study designs. While most were observational, two primary studies were randomised controlled trials, and one was a randomised controlled trial follow-up study. Consequently, the majority were susceptible to confounding factors and selection bias and had limited ability to infer causality. However, an advantage of the retrospective studies is the ability to select those born within the gestational age of interest. These studies also varied widely in sample size (from 60 to 278,508) and geographic setting, complicating direct comparisons between studies, but increasing generalisability.

#### Exclusion criteria and control/reference groups

The congenital adrenal hyperplasia studies (Table 2) typically had minimal exclusion criteria, primarily based on participant consent; however, the preterm birth studies (Table 3) applied more variable exclusion criteria. For example, Lee et al. [41] had no exclusion criteria, whereas Braun et al. [31] had extensive exclusion criteria. The latter also excluded “other fetal or [parental] diseases”; however, no details were provided, making it unclear which additional conditions were considered. While comprehensive and well-defined criteria are important for minimising confounding, it is equally important not to exclude key populations. For example, excluding those with pre-eclampsia and related signs (placental insufficiency or pathological Doppler findings) may limit generalisability, as pre-eclampsia is linked to preterm birth [43]. Notably, all four studies that excluded these populations [26, 27, 30, 31] reported negative overall outcomes following the use of antenatal corticosteroids, suggesting that they may have excluded those most likely to benefit. However, these studies are based on two datasets and two selected only term-born individuals [26, 27], so inferences should be made with caution. Control/reference group selection also differed. In the congenital adrenal hyperplasia studies, comparisons were often performed between affected and unaffected siblings. Moreover, multiple preterm birth studies used gestational age-matched reference groups, rather than pregnancies diagnosed at risk of preterm birth without antenatal corticosteroid administration; a comparison that would have been more appropriate, but likely more difficult to obtain.

#### Treatment protocols

Within the congenital adrenal hyperplasia studies (Table 2), there were limited data on the method and timing of mutation analysis (how/when the condition is tested for), treatment start dates and durations, and prenatal dexamethasone dosage (should be 20 µg/kg/day), restricting the investigation of these factors. However, the data that were available showed high similarity between studies, likely due to the majority being conducted within a single country (Sweden). On the other hand, the preterm birth studies (Table 3) mostly specified the use of 2 × 12 mg betamethasone administered intramuscularly 24 h apart. Variations from this protocol were more common in earlier datasets when less evidence about antenatal corticosteroid use and dosage was available (e.g. [36], a 50-year follow-up study). In addition, several studies did not state the formulation of antenatal corticosteroids used (phosphate versus acetate).

#### Consideration of sex in the study design

Research on congenital adrenal hyperplasia has more consistently explored sex-specific outcomes. However, even in these studies, particularly those conducted earlier, there was an inconsistent and unclear use of the terms “gender” and “sex”. In preterm birth studies, sex was often a covariate or subgroup rather than a primary variable of interest. Additionally, sex determination methods were rarely reported in these preterm birth studies, with most likely relying on genital appearance at birth. This could potentially lead to rare mis-classifications, particularly in individuals with disorders of sexual development.

#### Outcomes measured

The congenital adrenal hyperplasia studies (Table 2) primarily focused on long-term cognitive outcomes, reflecting concerns over the neurodevelopmental effects of prenatal dexamethasone exposure, particularly in those unnecessarily treated. In contrast, the preterm birth studies (Table 3) tended to focus on mortality, respiratory morbidity and anthropometric outcomes. However, six studies investigated other outcomes, each of which differed. While this broadens the scope of findings, it disallows comparisons for these outcomes.

#### Additional considerations

Other key considerations to the congenital adrenal hyperplasia studies (Table 2) include the use of American (not Swedish) norms, lack of correction for multiple analyses, and missing data for some individuals.

The preterm birth studies (Table 3) also often lacked key information about participants, including the number of fetuses and gestational age. Most studies included both singletons and multiples, but five did not specify. Separating data by the number of fetuses is important to avoid masking potential patterns. For example, Cheung et al. [44] reported that twins had lower mortality compared to gestational age-matched singletons between 29 and 37 weeks, but higher mortality after 38 weeks. Despite this, only one study [45] accounted for this distinction when analysing both singletons and twins. The ranges of gestational age investigated vary from 25 + 0–27 + 6 weeks to ≥ 40 weeks (most focused on preterm births), with an even distribution across the WHO definitions of preterm gestational age categories. However, there were inconsistencies/absences in the categorisation of gestational age, a lack of data for gestational age at delivery (three studies), and a lack of data for gestational age at administration (multiple studies).

To minimise confounding by birth or fetal growth restriction, many of the included preterm birth studies either excluded infants who were small-for-gestational-age or accounted for birth weight (or related measures) as a potential confounder in their analyses. However, reporting was inconsistent across studies. For example, Ramos-Navarro et al. [42], included intrauterine growth restricted offspring, but did not report whether there were group differences in growth restriction or adjust for it in their analyses. Additionally, two studies both reported significant differences in birth weight between groups, but did not specify whether any participants were small-for-gestational-age [46, 47]. Similarly, Battarbee et al. [33], as secondary analysis, did not report small-for-gestational-age status, despite this being included in the primary study.

The preterm birth secondary analysis studies (Table 3) also carried additional limitations inherited from their respective primary trials, including lack of adjustment for covariates, reliance on self-reported data subject to recall bias and methodological constraints, such as outdated gestational age measurement techniques or use of minimally active placebos. Other considerations amongst the preterm birth risk studies include low treatment compliance rates and low follow-up rates, both of which are common challenges in many studies.

#### Preterm birth systematic review

The systematic review [25] exclusively included randomised controlled trials, offering high internal validity. Randomised controlled trials are the gold standard in clinical research because randomisation reduces both selection bias and confounding factors. Using a single study design improves consistency and generalisability compared to observational studies; however [48], noted that populations in randomised control trials often differed from the target patient population in age, gender, race, health status, education, and socioeconomic status.

Moderate heterogeneity was observed for respiratory distress syndrome and severe intraventricular haemorrhage; however, sensitivity analyses revealed no significant differences in sample size, blinding or statistical models, suggesting that clinical heterogeneity was the likely cause (e.g., variability in populations or interventions). Nonetheless, this moderate heterogeneity does not undermine the overall conclusions. The limited data prevented assessment of heterogeneity for other outcomes.

The study utilised gestational age at entry and gestational age at antenatal corticosteroid administration interchangeably, although this assumption is not explicitly stated. It also reported a 2.9-week mean time between antenatal corticosteroid administration and delivery and cited evidence suggesting that antenatal corticosteroid administration within 24 h to 7 days before birth was necessary for beneficial outcomes, whereas antenatal corticosteroid administration more than 14 days prior may increase risks [49, 50]. Given this, the authors noted that many infants in the studies may not have benefited from antenatal corticosteroids, and differences in the proportions of sexes born within the beneficial interval could have influenced the results. Missing gestational age at entry and delivery data from three and four studies, respectively, further limited the analysis. Moreover, the absence of data on the proportion of term-born infants restricted analysis of outcomes in the target preterm population and prevented comparisons between preterm and term outcomes. Additional limitations included a small number of randomised control trials, the inability to investigate publication bias, and a gap of over 30 years between the earliest and most recent trials included.

## Discussion

The evidence from the congenital adrenal hyperplasia and preterm birth studies highlights the potential for sex-specific antenatal corticosteroid-related outcomes.

### Congenital adrenal hyperplasia

For congenital adrenal hyperplasia, the seven studies (Table 2) provided valuable evidence on sex-specific outcomes following prenatal dexamethasone. Females demonstrated consistent associations with negative cognitive outcomes, whereas cognitive findings for males were more heterogenous. Both sexes demonstrated sex-specific neurostructural differences; however, the significance of these findings remains unclear. Behavioural changes in males were also found, with similarly unclear clinical significance.

Females were associated with primarily adverse outcomes, including decreased general intelligence, executive function and mental processing outcomes, with more effects observed with longer treatment. Interpretation of the cognitive findings in females is complicated by contrasting results, for example those between Meyer-Bahlburg et al. [37] and Wallensteen et al. [22] for recall of heard numbers in non-congenital adrenal hyperplasia individuals. A potential explanation for such findings is differences in study design. If Wallensteen et al. [22] conducted both forward and backward components of the span board test (unclear due to lack of separate forward and backwards scores), it could suggest that prenatal dexamethasone in non-congenital adrenal hyperplasia females impairs the backwards recall of numbers more than it does forward. In support of this, only the forward component of the span board test showed a significant deficit, indicating that working memory may not be consistently affected in exposed females. Another possible explanation is the age differences between the populations (Table 2), however side effects noted in non-congenital adrenal hyperplasia dexamethasone children have not been confirmed at adult age [21]. Additionally, insufficient study power, selection bias, and differences in treatment regimens could have contributed to the contrasting findings. Meyer Bahlburg et al. [37] noted that some females with congenital adrenal hyperplasia received shorter durations of prenatal dexamethasone than females without the condition, which may have also influenced the outcomes and affected interpretations.

Significant findings were also seen in males, including changes in behaviour, brain structure and cognitive test results. The increased level of neutral behaviours in non-congenital adrenal hyperplasia dexamethasone males [20], were not explicitly classified as harmful by the authors, however, the study concluded that, because of the combination of these changes and prior cognitive concerns from other studies, prenatal dexamethasone may negatively affect fetal neurodevelopment in males.

Some beneficial male outcomes were also reported, but their specific link to prenatal dexamethasone is uncertain. Meyer-Bahlburg et al. [37] proposed that an improvement in cognitive outcomes (in both congenital adrenal hyperplasia affected and non-affected) caused by prenatal dexamethasone was unlikely and hypothesised that the results could be due to selection bias towards higher parental cognitive ability, despite no significant differences in parental IQ and education levels. Additionally, the Karlsson et al. [24] finding of a significant interaction between prenatal dexamethasone and sex for vocabulary within non-congenital adrenal hyperplasia populations also points towards improvement in cognitive outcomes in males. Separately, in both sexes, effects were not statistically significant, however, the study hypothesised that the non-significant changes may have resulted from insufficient power to detect true effect sizes.

Although there was no evidence of significant changes in mood or cognition in adults after prenatal dexamethasone exposure [21](Table 2), sex-specific structural brain changes (measured via MRI T1, T2 & diffusion-weighted imaging) were found, and thus their clinical significance remains unclear. As such, the study suggested that prenatal dexamethasone treatment may be associated with an increased risk in pregnancies.

Although all studies suggest that sex-specific antenatal corticosteroid-related outcomes exist for congenital adrenal hyperplasia, the strength of this evidence should not be overstated, given the considerations previously discussed (e.g., low geographical heterogeneity and incomplete data sets). In particular, the likely inclusion of some individuals in multiple studies, may lead to overrepresentation of findings and should be considered when examining the available data.

Furthermore, a key gap in current research is the limited variety of outcomes measured, with sparse evidence for somatic factors. Although not included in this review, some studies have explored outcomes beyond neurodevelopment/cognition, including metabolic function, with some evidence suggesting adverse impacts on lipid profile and glucose homeostasis [51].

Prenatal dexamethasone use for congenital adrenal hyperplasia remains controversial. Ethical concerns arise from the surmounting evidence indicating impacts on cognition, behaviour and brain structure, as well as exposure prior to confirmation of diagnosis or sex, since treatment must be initiated before 6–8 weeks of gestation. At the same time, dexamethasone is administered to reduce virilisation in affected female fetuses, a key potential benefit that must be considered alongside these risks, particularly given the ethical frameworks in many European countries restricting genital surgery without the child’s consent outside of medical emergencies [52]. However, evidence that non-dexamethasone-treated females with congenital adrenal hyperplasia may not have significantly higher rates of gender dysphoria [53], further complicates this risk-benefit balance. These considerations call into question the need for virilisation prevention and the morality of further prospective studies. If future research is pursued, it should follow the guidelines from The Endocrine Society or utilise retrospective data (to avoid exposing additional individuals to treatment). Greater understanding of the sexually-dimorphic responses to antenatal corticosteroids may instead be more ethical and clinically valuable in the widespread context of preterm birth treatment.

### Preterm birth risk

Antenatal corticosteroids for the risk of preterm birth are widely utilised and confer clear clinical benefit. The 21 studies (Table 3) [25] provide a useful starting point for investigating sexually-dimorphic outcomes following antenatal corticosteroids for preterm birth risk, however findings are often contradictory and interpretations limited.

There are strong and consistent evidence that antenatal corticosteroids reduce both perinatal and neonatal mortality, as shown in numerous studies and confirmed by large systematic reviews, including [54]. In this context, the conflicting findings regarding overall mortality rates in the primary and secondary studies are somewhat unexpected and remain difficult to fully explain. These discrepancies may stem from a subset of the literature that selected sex-stratified outcomes. Additionally, differences in study characteristics (e.g., exclusion criteria, adjustment for confounding factors, and how sex-specific effects were assessed) may have contributed.

One potential contributor to this variability is fetal growth restriction, an important risk factor for adverse offspring outcomes that should be considered alongside preterm birth risk [55, 56]. While some studies in the literature do not clearly differentiate between preterm birth and fetal growth restriction, studying all infants born less than a specific weight threshold, none of the studies included in the preterm birth risk section of this review did as such. To reduce potential confounding by growth restriction, many of the included studies either excluded infants who were small-for-gestational age or intrauterine growth restricted (< 10th percentile), or assessed group differences and adjusted for birth weight (or similar variables) in their analyses. However, reporting was incomplete in several studies. Consequently, although most studies attempted to account for this, uncertainty remains regarding the extent of residual confounding.

Beyond confounding, differences in how sex-specific effects were assessed may also have contributed to variability in findings. This highlights the importance of comparing exposed and non-exposed individuals within each sex, rather than directly comparing males and females, which may be confounded by baseline sex differences in mortality and morbidity (the ‘male disadvantage’). This approach would help improve comparability and allow for a more accurate assessment of treatment effects. Additionally, 15 studies identified sex-specific antenatal corticosteroid-related outcomes; of the six studies specifically designed to investigate sex differences, five identified such effects, possibly suggesting that targeted study designs may increase the likelihood of detecting sex-specific effects.

In addition, the differences in the primary ALPS study [32] and secondary analysis [33] findings likely stem from variations in study populations (e.g., exclusion of late preterm births and major congenital abnormalities in the secondary study, reducing the sample by ~ 500) and primary outcomes (the original study used a composite of respiratory support, stillbirths and neonatal deaths, whereas the secondary study used a composite of various severe neonatal morbidities). Stricter thresholds for the primary outcomes (< 0.048 vs. <0.05) and effect modification (< 0.05 vs. <0.1) likely contributed.

Across the included primary and secondary studies (Table 3), male sex was frequently associated with worse outcomes (mortality and morbidity rates) following antenatal corticosteroids compared to females. However, it remains unclear whether this reflects a sex-specific effect of antenatal corticosteroid exposure or the higher baseline risk in preterm males (i.e., irrespective of treatment). In contrast, the systematic review by [25] did not demonstrate this. These primary and secondary studies also found that term-born females had poorer outcomes compared with males, and antenatal corticosteroids may have greater effects on anthropometric values in females; these outcomes were not investigated in the systematic review.

The systematic review did, however, find sex differences concerning antenatal corticosteroid type, reporting that dexamethasone was only effective in reducing respiratory distress syndrome rates in females, and betamethasone only being effective in males. No statistical comparison of outcomes between males and females within each corticosteroid group was performed, which would have further strengthened the evidence for sexually dimorphic effects. Notably, the two largest studies included in the systematic review reported conflicting results, one showing benefits of antenatal corticosteroids in females, but not males, and the other the opposite. These inconsistencies raise questions about potential modifying factors, e.g., gestational age and birth weight. In addition, all studies involving betamethasone reported significant benefits for males only, whereas dexamethasone reported significant benefits for females only. Evaluation of potential associations between antenatal corticosteroid type and sex-specific outcomes was not feasible based on the available primary and secondary study data.

The systematic review also suggested that sex differences in survival may be more apparent in extremely or very early preterm infants, potentially explaining the lack of differences observed, given that the average gestational age at delivery was ~ 32–35 weeks. The primary and secondary data may support the idea that sex differences in survival vary by gestational age, as the three studies that reported sex differences in mortality focused only on infants born at either < 32 weeks or > 37 weeks ([40] reported sex differences only in term infants [41], focused only on extremely preterm births, and [42] focused on infants born at < 32 weeks).

The systematic review studies had earlier datasets, fewer differences in sample sizes and greater variation in administration regimens compared to the primary and secondary studies. These differences may also have contributed to variations among findings.

The current evidence on sex differences following treatment for the risk of preterm birth is limited, with only one systematic review [25] (based on studies all older than 15 years) and a collection of highly heterogeneous primary and secondary studies (Table 3). Additionally, the low proportion of primary studies included (21/175) underscores the need for changes in current research practices to ensure that sex differences are consistently investigated. There is insufficient evidence to suggest any patterns between sex-specific antenatal corticosteroid-related outcomes and morbidity rates excluding respiratory conditions, gestation number, data-set country, APGAR scores and antenatal corticosteroid regimens.

Further research is required to clarify whether male sex remains a risk factor following antenatal corticosteroids, whether females are at greater risk if they are born at term, and whether antenatal corticosteroid types have varying efficacy across the sexes. Ethical restraints mean that new randomised controlled trials with untreated control groups should not be conducted. If the sex-specific efficacy of corticosteroid type is confirmed, this would warrant changes in standard clinical practice so that treatment is tailored to fetal sex. In parallel, research into the physiological mechanisms of parturition and optimising preterm birth predictors (such as corticotropin-releasing hormone, where early increases correlate with an increased risk of preterm birth) [57] could reduce unnecessary corticosteroid use.

### Potential mechanisms underlying sex differences

Human and animal model studies can be utilised to propose possible mechanisms behind sex-specific antenatal corticosteroid-related outcomes. Including a study which investigated the effects of inhaled corticosteroids to control asthma symptoms in asthmatic pregnancies [58].

#### Differential HPA axis activity & placental factors

In guinea pigs, interactions between sex steroids and the HPA axis were observed following antenatal corticosteroids. At ~ 75 days, male adult offspring had increased testosterone and reduced HPA axis activity, but by ~ 150 days, these levels normalised [59, 60]. These findings suggest that testosterone plays a role in HPA axis suppression in males following antenatal corticosteroid exposure. Liu et al. [59] also reported that female offspring had higher cortisol levels (both basal and stimulated) in the follicular and early luteal phases, but lower cortisol levels in the late luteal phase following antenatal corticosteroids. This evidence from experimental animals highlights the potential impact of antenatal corticosteroids on HPA axis regulation.

Alterations in the limbic system, which is rich in glucocorticoid receptors and involved in HPA axis feedback regulation, may contribute to heightened stress reactivity in females (Table 3). In vitro studies have shown that glucocorticoid exposure reduces neural progenitor cell proliferation and induces long-lasting DNA methylation changes in hippocampal progenitors [61, 62]. Additionally, Dean & Matthews [63] reported that, unlike males, female guinea pig offspring exposed to antenatal corticosteroids presented increased mRNA and levels of glucocorticoid and mineralocorticoid receptors in the hippocampus (where glucocorticoids can bind to both receptor types). If similar sex-specific changes in glucocorticoid and mineralocorticoid receptor expression occur in peripheral tissues involved in metabolism (e.g., liver or adipose tissue), this could help explain the interaction between sex and rates of overweight in offspring following antenatal corticosteroid treatment (Table 3).

Lower HPA axis activity in exposed males may also impair blood pressure regulation (e.g., lower normetanephrine and umbilical endothelin-1) [29], leading to cardiovascular instability and dysfunction. This may explain the higher severe intraventricular haemorrhage and mortality rates in males following antenatal corticosteroids. Increased cortisol in females may help explain their greater susceptibility to anthropometric changes and could also provide a potential mechanism for the higher rates of mental disorders (e.g., anxiety) observed following antenatal corticosteroids in those at risk of preterm birth (and those with autoimmune or inflammatory disorders) [64], although no sex-specific differences were reported in that study.

Although synthetic corticosteroids largely bypass metabolism by 11beta-hydroxysteroid dehydrogenase type 2, sex-specific differences in placental enzyme activity have been reported following corticosteroid use [58, 65]. Stark et al. [65] reported that, compared with males, female infants born < 72 h after betamethasone administration had higher 11beta-hydroxysteroid dehydrogenase type 2 activity and umbilical cortisol, but no significant sex differences were detected in those born > 72 h after betamethasone administration. The authors hypothesised that females auto-regulate 11beta-hydroxysteroid dehydrogenase type 2 activity, limiting adrenal suppression and leading to increased HPA axis activity. Similarly, Murphy et al. [58] reported increased 11beta-hydroxysteroid dehydrogenase type 2 activity in female fetuses from asthmatic pregnancies using inhaled corticosteroids.

Other placental factors, such as glucocorticoid receptor expression and placental dysfunction, may also play a role. Bhaumik et al. [66] reported increased levels of the glucocorticoid receptor ⍺-C (which promotes apoptosis) in females and the glucocorticoid receptor β (which induces glucocorticoid resistance) in males. Thus, sex-divergence in receptor expression may contribute to greater growth reduction in females. In addition, males are at a greater risk of placental insufficiency than females [67], reducing 11beta-hydroxysteroid dehydrogenase type 2 expression and activity. It may therefore modify antenatal corticosteroid efficacy, despite most synthetic corticosteroid being metabolised, and could theoretically be a mechanism contributing to sex-specific differences. However, several of the included studies excluded pregnancies complicated by placental insufficiency.

Overall, available evidence suggests HPA axis activity and placental factors between sexes following exposure to antenatal corticosteroids, providing potential mechanisms underlying sex-specific differences. However, conflicting findings exist. For example, the effects of antenatal corticosteroids on mineralocorticoid receptor hippocampal expression in male guinea pigs are inconsistent; [63] reported no changes, whereas [59] and [60] reported increases.

What is clear is that the relationships among antenatal corticosteroids, HPA axis activity and placental factors are complex and influenced by several factors, including timing of antenatal corticosteroid administration and assessment age. Furthermore, findings from experimental animal and in vitro studies may have limited applicability to humans.

#### Differential prior risk of respiratory distress syndrome

Evidence from rats suggests that males have lower oestrogen receptor beta levels in the fetal lung epithelium, leading to decreased Na+ transporter mRNA and protein expression when compared to females. As a result, less lung fluid reabsorption occurs and greater volumes of lung liquid are present at birth in male newborns [68]. Additionally, testosterone produced by the testes has been shown to delay surfactant production in males [69], which would increase lung surface tension, increase the alveolar “wetness”, increase the work of breathing, with consequences for impaired gas exchange, and the risk of atelectasis and pulmonary oedema. Combined, the lower oestrogen receptor beta levels and greater testosterone production in males compared to females may therefore explain the greater risk of respiratory distress and increased morbidity observed in males irrespective antenatal corticosteroid administration.

## Overall conclusions

**Fig. 3:**
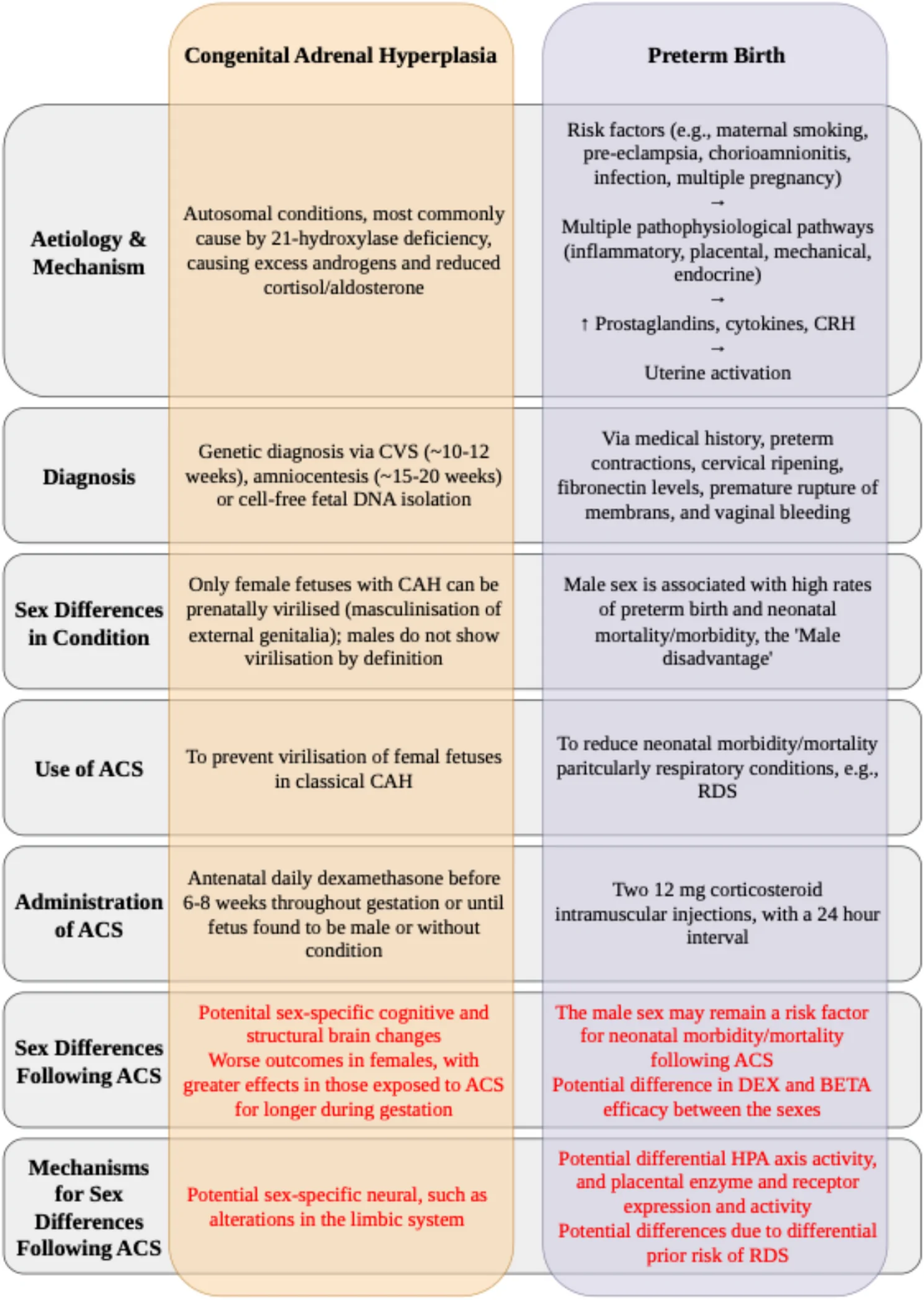
**Overview of congenital adrenal hyperplasia and preterm birth risk with antenatal corticosteroids and sex-specific outcomes.** The figure presents an expanded overview of congenital adrenal hyperplasia and preterm birth risk across key domains, incorporating potential sex-specific outcomes following antenatal corticosteroids and the mechanisms behind them. Red text highlights the focus of this review. Antenatal dexamethasone was historically administered daily from 6–8 weeks of gestation throughout gestation (or until fetus was found to be unaffected or male); however, this is not currently recommended in clinical practice [2].11β-HSD2, 11beta-hydroxysteroid dehydrogenase type 2; ACS, antenatal corticosteroids; BETA, betamethasone; CAH, congenital adrenal hyperplasia; CVS, chorionic villus sampling; DEX, dexamethasone; HPA axis, hypothalamic-pituitary-adrenal axis; RDS, respiratory distress syndrome.

Figure 3 provides an overview of antenatal corticosteroids and their sex-specific outcomes in the context of congenital adrenal hyperplasia and preterm birth risk.

In conclusion, current evidence suggests that sexually-dimorphic offspring outcomes may follow antenatal corticosteroid administration for the treatment of congenital adrenal hyperplasia and preterm birth risk. While ethical and clinical risks limit future research in the context of congenital adrenal hyperplasia, the preterm birth studies highlight the importance of studying sex differences and their underlying mechanisms. Studies undertaken highlight the role of differences in placental enzyme and receptor expression and activity, fetal HPA axis function, and respiratory complications as putative mechanisms underlying sex differences. However, conflicting evidence persists, and most studies rely on non-primate animal models, which differ from humans in multiple aspects of prenatal development and endocrine axes [70–72].

Studies are necessary to explore these findings further and to clarify additional mechanisms, including the processes underlying sex-specific outcomes in term-born infants and across different types of antenatal corticosteroids. Understanding the magnitude of these effects and mechanisms involved is key for developing a personalised medicine approach and improving both the short- and long-term health outcomes for the next generation of both sexes.

## Data Availability

All data generated or analysed during this study are included in this published article.

## Abbreviations

11β-HSD2: 11beta-hydroxysteroid dehydrogenase type 2
ACS: Antenatal corticosteroids
AD: Axial diffusivity
ALPS: Antenatal Late Preterm Steroids study
APGAR: Appearance, Pulse, Grimace, Activity and Respiration score
AST: Auckland Steroid Trial
BETA: Betamethasone
BL: Birth length
BPD: Bronchopulmonary dysplasia
BW: Birthweight
CAH: Congenital adrenal hyperplasia
cfDNA: Cell-free fetal DNA
CS: Corticosteroid(s)
CVS: Chorionic villus sampling
DEX: Dexamethasone
DNAm: DNA methylation
FA: Fractional anisotropy
fFN: Fetal fibronectin
FMBR: Finnish Medical Birth Register
GA: Gestational age
GC: Glucocorticoid(s)
GR: Glucocorticoid receptor
HPA axis: Hypothalamic–pituitary–adrenal axis
IM: Intramuscular
IUGR: Intrauterine growth restriction
IVH: Intraventricular haemorrhage
KI-GRB: Karolinska Inventory of Gender Role Behaviour
M1/M0: Moment ratio (measurement of gas-mixing efficiency)
MR: Mineralocorticoid receptor
NEPSY: NEuroPSYchological Assessment
NICU: Neonatal intensive care unit
PROM: Premature rupture of membranes
RCT: Randomised control trial
RDS: Respiratory distress syndrome
SGA: Small-for-gestational-age
WHO: World Health Organisation
